# Influence of the sebaceous gland density on the stratum corneum lipidome

**DOI:** 10.1038/s41598-018-29742-7

**Published:** 2018-07-31

**Authors:** Matteo Ludovici, Nina Kozul, Stefano Materazzi, Roberta Risoluti, Mauro Picardo, Emanuela Camera

**Affiliations:** 1 Laboratory of Cutaneous Physiopathology, San Gallicano Dermatological Institute IRCCS, Rome, Italy; 2 Department of Chemistry, University of Rome “Sapienza”, Rome, Italy

## Abstract

The skin surface lipids (SSL) result from the blending of sebaceous and epidermal lipids, which derive from the sebaceous gland (SG) secretion and the permeability barrier of the stratum corneum (SC), respectively. In humans, the composition of the SSL is distinctive of the anatomical distribution of the SG. Thus, the abundance of sebum biomarkers is consistent with the density of the SG. Limited evidence on the influence that the SG exerts on the SC lipidome is available. We explored the differential amounts of sebaceous and epidermal lipids in areas at different SG density with lipidomics approaches. SC was sampled with adhesive patches from forearm, chest, and forehead of 10 healthy adults (8F, 2M) after mechanical removal of sebum with absorbing paper. Lipid extracts of SC were analysed by HPLC/(-)ESI-TOF-MS. In the untargeted approach, the naïve molecular features extraction algorithm was used to extract meaningful entities. Aligned and normalized data were evaluated by univariate and multivariate statistics. Quantitative analysis of free fatty acids (FFA) and cholesterol sulfate (CHS) was performed by targeted HPLC/(-)ESI-TOF-MS, whereas cholesterol and squalene were quantified by GC-MS. Untargeted approaches demonstrated that the relative abundance of numerous lipid species was distinctive of SC depending upon the different SG density. The discriminating species included FFA, CHS, and ceramides. Targeted analyses confirmed that sebaceous FFA and epidermal FFA were increased and decreased, respectively, in areas at high SG density. CHS and squalene, which are biomarkers of epidermal and sebaceous lipid matrices, respectively, were both significantly higher in areas at elevated SG density. Overall, results indicated that the SG secretion intervenes in shaping the lipid composition of the epidermal permeability barrier.

## Introduction

Skin is a tissue exceptionally enriched in lipids. The involvement of skin lipids in the maintenance of skin and entire body homeostasis is well documented^1,2^. Additionally, disturbance of the skin lipid arrangement is linked to several skin diseases such as acne, atopic dermatitis, and psoriasis, to mention the main ones^3–6^. The skin surface lipids (SSL) result from the mixture of lipids deriving from the two main cutaneous lipid sources. Sebum, an amorphous lipid matrix secreted by the sebaceous gland (SG), and epidermal lipids in the stratum corneum (SC), represent the main lipid compartments at the skin surface^7^.

SC covers the entire body surface, even though features of the SC such as thickness and hydration change considerably from site to site. The density of the SG is particularly high on the scalp and foreheads, where it reaches 400–900 glands/cm^2^. In contrast, limbs show a considerably lower SG density^8^. Abundance of sebum is consistent with the SG density although sebometry and follicle number are not linearly correlated^9,10^. Sebum and SC have a remarkably diverse and unique lipid composition. Sebum is an oily to waxy mixture composed by 30–60% triglycerides (TG), 20–30% wax esters (WE), 10–30% FFA, and 10–20% squalene, as weight/weight percent (w/w %)^11^. Squalene is specific to human sebum where it is present at exceptionally high concentrations compared to other body lipid compartments. Minor components of sebum are cholesterol that together with sterols esters accounts for the 2–4%, and diglycerides (1–2%)^10,12^. The epidermal lipid compartment localized in the SC contains approximately 50% ceramides, 15% FFA, and 25% cholesterol and cholesterol sulfate (CHS)^6,13,14^. The latter one is present at a fairly high concentration in the SC wherein it regulates cohesion and desquamation. Extrusion of CHS and amounts of FFA take part to the regulation of skin pH^15^. Altogether, lipids account for the 10% of the SC weight. In the SC, the lipid and protein matrices are tightly intertwined to form the skin permeability barrier (SPB)^16^. Formation of a competent SPB is a complex process wherein several lipid biosynthetic pathways concur in the build-up of the lipid barrier. The epidermal sphingolipid metabolism leads to the biosynthesis of broad spectra of ceramides that are key regulators of the epidermal differentiation and inflammation^17^. The ceramide families found in the SC present very long chain FA, normally longer than 20 carbons, bound to the sphingoid bases. Moreover, ceramides bearing hydroxylated FA are partly transformed into acylceramides upon binding to long chain FA^18,19^. Little is known about the influence of the SG product on the epidermal lipid arrangement. It has been suggested that sebum secreted by the SG contributes to the ‘self-sterilizing’ properties of human skin^20^. Sapienic acid (FA C16:1n-10) is a sebum specific FA and the most active antimicrobial skin surface lipid^21^. Moreover, sebaceous FFA participate to the net value of superficial pH^22^. Thus, abundance and quality of sebum might influence the biochemical environment and the resident flora on the skin surface. Moreover, sebum can be regarded as a surface active biofluid that could impact physical properties of the SC.

The diversity of skin lipids together with the different sources of their production pose a challenge for their qualitative and quantitative assessments. Lipidomics has gained importance in several fields of bioscience and has enhanced its potential in deciphering the complexity of the lipid composition in different biological matrices^23^. Conditions of suitable lipidomics approaches depend on the lipid source and the specific problem of interest. Lipidomics methods include well established separation and detection techniques such as thin layer chromatography (TLC), liquid chromatography (LC), gas chromatography (GC), mass spectrometry (MS)^24,25^. Approaches to investigate the two major skin lipid sources have been designed for both sebum and epidermal lipids^12,18,26–35^. A shotgun MS-based lipidomic approach has been presented that addressed the comprehensive identification and profiling of ceramide classes, TG, and cholesterol esters (CE) in human SC^36^. Intense research devoted to the characterization of sebaceous lipids by GC-MS dates back to the last century. More recent GC-MS methods have widened the lipid class coverage compared to the traditional ones^29^. Whereas, with the progresses achieved with LC-MS, investigations of SPB lipids have produced overwhelming analytical approaches to address sphingolipids^18,30–34^. In LC-MS, electrospray ionization (ESI) is the most commonly used ionization technique. The ESI in negative ion mode ((-)ESI) is a preferable modality to detect selectively lipid species that tend to form deprotonated ions^26^. In the case of SSL, (-)ESI mode allows detecting predominantly the lipids of the SC whereas it is unfavourable for the detection of neutral lipids enriching sebum^12^. In contrast, FFA are detected in the same ionization condition irrespective to their epidermal or sebaceous origin. With the exception of FFA, due to the preferential detection of SC lipids under negative ionization modes, this strategy was exploited to gain insights in the relationship between lipid composition of the SC and the anatomical distribution of the SG. Nevertheless, FFA of the sebaceous type and of the epidermal type were discriminable based on the knowledge of the different predominant chain length and unsaturation pattern presented in the two compartments. To investigate the lipidome of SC lipid extracts from areas at different SG density, SC was sampled mechanically from forearms, chests, and foreheads by tape-stripping. These sites present, in the order, increasing density of the SG. Untargeted approaches associated with multivariate analyses were employed to evidence the changes of the epidermal lipids in areas with different SG density. The present results open new perspectives in the investigation of the role of SG in the SC homeostasis and dysfunction.

## Materials and Methods

### Chemicals, reagents and reference compounds

HPLC/MS-grade methanol, isopropanol, acetonitrile, chloroform, and acetone were purchased from Merck (Darmstadt, Germany), HPLC/MS-grade ammonium formate (HCOONH_4_) was purchased in granular form from Fluka (Buchs SG, Switzerland), and HPLC/MS-grade formic acid (HCOOH) was purchased from Sigma-Aldrich (Milan, Italy). Authentic palmitoleic acid (MW 254.4), oleic acid (MW 282.5), linoleic acid (MW 280.4), α-linolenic acid (MW 278.4), 11Z,14Z eicosadienoic acid (MW 308.5), 5Z,8Z,11Z eicosatrienoic acid (MW 306.5), arachidonic acid (MW 304.5) and 5Z,8Z,11Z,14Z,17Z eicosapentaenoic acid (302.5) and palmitoleic acid D14 (d14-PoA, MW 268.4) were purchased from Cayman Chemical (Ann Arbor, MI, USA). Whereas, lauric acid (MW 200.3), tridecanoic acid (MW 214.3), myristic acid (MW 228.4), myristoleic acid (9c) (MW 226.4), pentadecanoic acid (MW 242.4), palmitic acid (MW 256.4), palmitoleic acid (MW 254.4), margaric acid (MW 270.4), 10c-heptadecenoic acid (MW 268.4), stearic acid (MW 284.4), oleic acid (MW 282.5), nonadecanoic acid (MW 298.5), arachidic acid (MW 312.5), 11c-eicosenoic acid (MW 310.5), heneicosanoic acid (MW 326.6), behenic acid (MW 340.6), erucic acid (MW 338.6), tricosanoic acid (MW 354.6), lignoceric acid (MW 368.6), nervonic acid (MW 366.6), pentacosanoic acid (MW 382.7), cerotic acid (MW 396.7) were purchased from Larodan (Malmo, Sweden). Cholesterol sodium sulfate (MW 488.7) was purchased from Sigma-Aldrich (Milan, Italy), while deuterated cholesterol sulphate sodium salt (d7-CHS, MW 495.3) and deuterated cholesterol (d6-cholesterol, MW 392.7) were purchased from CDN Isotopes (Pointe-Claire, Quebec, Canada). N-palmitoyl-d31-D-erythro-sphingosine (d31-Cer16:0, MW 569.1) was purchased from Avanti Polar Lipids (Alabaster, AL, USA). Ceramides, with hydroxyl and non-hydroxy acyl groups were purchased from Matreya (Pleasant Gap, PA, USA). Stock solutions of the standard lipids were prepared in ethanol or in choloroform/methanol 2:1 according to the manufacturer’s instructions and then diluted in the mixture used to dissolve stratum corneum (SC) extracts, namely acetone/methanol/isopropanol 40:40:20.

### Human stratum corneum sampling

Stratum corneum (SC) was sampled from three different body sites of 10 Caucasian volunteers (8 females and 2 males, mean age 37.5 ± 7.6 years), by repetitive tape stripping with D-Squame^TM^ patches (CuDerm Corp., Dallas, TX, USA). Volunteers showed no signs of skin diseases in any of the sampled areas. SC was sampled from the volar surface of the forearms (ARM) and central area of the foreheads (HEAD) in both female and male panellists, whereas SC was sampled from the central area of chests (CHEST) only in women, due to the body hair present in men. The study was performed in agreement to the Helsinki Declaration after the approval by the Central Ethics Committee ‘Fondazione GB Bietti’, with the written informed consent obtained from each volunteer.

Four sampling discs were weighed before use. In detail, an area of the plastic strip holding four patches was cut with scissors and weighed. Similarly, an extra patch, used for the superficial SC removal, was cut together with the holding plastic foil. The steps of the SC sampling were: (1) Cigarette paper was applied onto to skin surface to remove the superficial sebum from the sampling area by absorption before SC sampling; the paper was pressed onto the skin with a 100 g weight that was rolled three times over the external surface of the paper. The cigarette paper was peeled off the skin and discarded. (2) To remove the very superficial SC, the predisposed single cleaning disc was applied to the sampling area and the 4 opposite points were marked with a dermographic pen. The patch was pressed onto the skin area for 10 seconds with a D-Squame pressure instrument (D500) providing a pressure of 225 g/cm^2^ and then removed off the skin surface with an angle of about 45 °C. This operation was repeated three times and the patch was discarded. Each of the four sampling patches predisposed for the sampling was used to strip three times the area devoid of the superficial SC, with the modality described above for the single cleaning patch. At the end of sampling, the collected patches were weighed together with the plastic support for the gravimetric assessment of the sampled SC. The patches adherent to the plastic support were wrapped in tin foils and stored at −80 °C until processing. Demographics and supplemental information are provided in the Supplementary Table S1.

### Sample preparation

Different solvent systems were tested in order to select the extraction method suitable for the extraction of lipids from the tapes. Extraction systems reported in the Literature were evaluated, such as choloroform/methanol 2:1, methyl-tert-butyl ether (MTBE), MTBE/methanol, and methanol only^30,37,38^. The first two systems had to be excluded as an apparent dissolution of the D-Squame^TM^ polymer occurred and limited the following analyses due to a severe contamination of the HPLC and MS systems. In contrast, methanol extraction provided performances consistent with previous reports^30^. The extraction processes was adapted in order to extract the patches from disposable labware. Moreover, to limit exposure to methanol, which poses some health hazard, we evaluated the extraction yield of absolute ethanol compared to methanol as previously investigated for sebum specimens^12,35^. In particular, 60 × 15 mm PE-LD Petri’s dishes (PD) (CELLSTAR, Greiner Bio-One GmbH, Frichenhausen, Germany) were employed for the purpose. Test patches obtained from the same volunteer in the adjacent areas on the forearm were transferred to the PD with clean tweezers and placed at the bottom of the PD with the adhesive surface upward. Extraction was carried out with 2 mL methanol or absolute ethanol. The PD were placed on a rocking tray at 4 °C and left 60 minutes under continuous shaking. The organic phases were collected into Eppendorf tubes and evaporated under nitrogen. The final lipid extracts were dissolved in 100 µL of acetone/methanol/isopropanol 40/40/20. On average, based on the weight of the sampled SC for each sample we obtained solutions of about 8 mg SC/mL. To test the efficacy of the two extraction methods, we compared TLC runs of SC extracted with the two methods. SC were separated and stained as previously reported^12^. No apparent differences were observed in the TLC analysis of the different lipids extracts (not shown). Due to the simplicity and reduced toxicity of the extraction solvent, ethanol was elected as the solvent system to be used in the following study. Moreover, the extraction of D-Squame^TM^ with absolute ethanol proved to be compatible with the HPLC/MS analysis of SC and provided an extraction yield comparable to methanol. The study samples were extracted after addition of a mixture of internal standards in order to normalize variability during the pre-analytical and analytical phases of the process. In particular, 100 µL of a solution of d14-PoA and d31-Cer16:0, 100, and 2 µM, respectively, and equal 10 µM concentration of d6-cholesterol and d7-CHS, in acetone/methanol/isopropanol 40/40/20 were added to the samples before extraction. The ethanol used for the extraction was transferred to an Eppendorf tube and evaporated to dryness under nitrogen. Before analyses, sample extracts were dissolved in 100 µL of acetone/methanol/isopropanol 40/40/20.

### Instrumentation

#### HPLC

The chromatographic apparatus consisted of a 1200 series rapid resolution HPLC (Agilent Technologies, Germany) equipped with a degasser, autosampler, and thermostated column compartment from the same manufacturer. For the rapid resolution reversed phase (RP) HPLC separation, the Kinetex C8, 50 × 2.1 mm, 1.7 µm, particle section, 100 Å pore section column with the SecurityGuard ULTRA Cartridge precolumn (Phenomenex, Castel Maggiore, BO, Italy), with maximal operational backpressure at 600 bar, were used. SC samples and authentic standards were eluted with a binary gradient of (A): 5 mM ammonium formate in water containing 20% of the methanol/isopropanol 95:5 mixture and (B): methanol/isopropanol 95/5. The mobile phases were filtered through 0.45 μm glass filters and continuously degassed under vacuum. The elution program was as follows: 0–6 min 60% B, 20 min 99% B, 20–30 min 99% B, 36 min 60% B, 36–38 min 60% B. A post-run of 2 min at 60% B was included. The flow rate was maintained at 0.4 mL/min during the entire HPLC run and post-run time (2 minutes). The column was thermostated at 60 °C. The injection volume was 4 μL. The injector needle was washed with the mobile phase in the wash port in the terminal part of the HPLC runs. The eluent outlet was connected to two different MS analyzers for detection and characterization.

#### Electrospray ion source and time of flight MS

Measurements of accurate mass and isotope pattern were conducted with a G6220A series TOF-MS (Agilent Technologies, Germany) equipped with an ESI interface operating in the negative ion mode. Analytes eluted from the LC system were introduced into the TOF-MS apparatus at the operating chromatographic flow rate (see chromatographic conditions). Nitrogen was used as the nebulizing and desolvation gas. The temperature and the flow rate of the drying gas were 350 °C and 10 L/min, respectively. The capillary and the cone voltage were 4,000 and 60 V, respectively. Scan mode TOF mass spectra were acquired in the negative ion mode by using the TOF at 10,000 mass resolving power for scans over the *m/z* range from 100 to 1200. MS scans were processed using the Mass Hunter software (B.01.03 version). To enhance accurate mass measurement for the ion species, a reference solution was vaporized in continuum in the spray chamber. The resulting data were converted to mass centroid from which the accurate *m/z* value was measured. Quantitative analyses of FFA and CHS was pursued with the same LC-MS method.

#### ESI-MS with a triple quadrupole MS

ESI tandem mass (MS/MS) spectra were obtained with a G6410A series triple quadrupole (QqQ) (Agilent Technologies, Germany). Data were acquired in the positive ion mode at unit mass resolving power by scanning ions between *m/z* 100 and 1200. MS spectra were averaged and processed with the Mass Hunter software. Analytes eluted from the LC system were introduced into the QqQ instrument at the operating flow rate (see chromatographic conditions). Nitrogen was used as the nebulizing and drying gas, and the source settings were the same used above with the TOF-MS. For the compound identification, precursor ion (precION) experiments were carried out by scanning the third quadrupole, while product ion (prodION) experiments were carried out by scanning the first quadrupole. The second quadrupole was used as collisional cell in both experiments. The collision energy applied was 34 V, while the fragmentor (F) voltage was set at 140 V.

#### GC-MS

Gas chromatography coupled to electron ionization MS selected ion monitoring (GC-MS) was employed to determine cholesterol and squalene quantitatively in the skin surface lipid extracts. Samples were analysed with a GC 7890A coupled to the MS 5975 VL analyser (Agilent Technologies, CA, USA). Quantitative analysis of cholesterol and squalene was performed by a GC-MS method as previously reported^11,39^. Briefly, 20 µL of the SC extract dissolved in 100 µL of acetone/methanol/isopropanol 40/40/20 were dried under nitrogen and derivatized with 100 µL BSTFA added with 1% trimethylchlorosilane (TMCS) in pyridine. To generate the trimethylsilyl (TMS) derivative of cholesterol, the reaction was carried out at 60 °C for 60 minutes^40^. GC separation was performed with the 30 m–0.250 mm (i.d.) GC DB-5MS UI fused silica column (Agilent Technologies, CA, USA), chemically bound with a 5% diphenyl - 95% dimethylpolysiloxane cross-linked stationary phase (0.25 mm film thickness). Helium was used as the carrier gas. Samples were acquired in single ion monitoring coupled to scan mode by means of electron impact (EI) MS. Calibrations curves were built against the deuterated IS d6-cholesterol and results were reported as total nmole of cholesterol and squalene.

### Data processing

#### HPLC-MS data extraction

Prior to the univariate and multivariate analyses, the data acquired by LC-MS were processed with the Mass Hunter Qualitative Analysis software (B.01.03 version). The collective LC-MS data were processed using the Molecular Feature Extraction (MFE) algorithm in the Mass Hunter Qualitative Analysis software. The naïve MFE algorithm finds the ion signals in the total ion chromatogram (TIC) by removing the chemical background and extracts peaks characterized by retention time (RT), *m/z*, ion intensity, and isotope distribution. Compound chromatograms were extracted from the TIC in association with pure spectra. This process contributed to the reduction of instrument noise and reduced the number of relevant features as described^41^. The number of final features generated was dependent upon the applied extraction filters, typically 100–1200 *m/z* mass range, selection of peak’s height, compounds counting, ion species (deprotonated ion, ion adducts, neutral losses), and charge state set to 1 with a peak spacing tolerance 0.0025 m/z, plus 20 ppm. MFE was also run in a targeted manner to search internal standards (IS) against their elemental formula. For each LC-MS data file, the MFE tool generated a list of all possible unknown and known components defined by RT, accurate mass (*m/z*), isotopic pattern, and abundance. The list was then converted into a compound extraction format (CEF) reporting the RT, *m/z*, and abundance of all meaningful species, among unknown and known species, including IS. The CEF files derived from data files generated by analysing each SC sample in duplicate were then exported into the Mass Profiler Professional package (MMP, software version 12.6.1, Agilent Technologies, CA, USA).

#### Data analysis and multivariate statistics

To interpret the processed data, statistical analyses and multivariate statistics were performed with the aid of specific tools within the MPP package. The CEF data files were cleaned of background noise and unrelated ions by the molecular alignment and filtering of data. All the available data (full scan mode from *m/z* 100 to 1200 and RT window 0.01 to 30 min) and minimum absolute abundance of 1000 counts were used to filter the data. Next step consisted of the alignment of the features across the SC samples. A tolerance window of 0.6 min and 20 ppm + 2 mDa were used to perform RT correction with labelled IS (d14-PoA and d31-Cer16:0) and *m/z* alignment, respectively. A total of 2661 entities were found in the entire set of analysed samples after alignment. Recursive analysis of original data files was performed by extracting the 2661 shared by all samples. Entities were filtered by frequency (those which appeared in 100% of samples in at least one group of samples were chosen). Normalization of the signal intensity of features was against intensity of d31-Cer16:0 in the respective sample. For baseline correction, all the compounds were treated equally regardless of their intensity. Such a correction subtracts the mean abundance of each entity from the corresponding values in each sample. Statistical significance analysis using the one way ANOVA and a level of probability of 0.001 was used as the criterion for significance. To individuate grouping due to similarities of the profiles of expression of lipid metabolites, we included principal component analysis (PCA), which is an unsupervised technique applied to explore the data and reduce the data dimension^42^. To delineate differential expression of lipid metabolites in the SC sampled from ARM, CHEST, and HEAD, volcano plots and hierarchical clustering were performed. Descriptive statistics and ANOVA followed by Tukey’s multiple comparisons test was performed on FFA profiles of abundance with the XLSTAT statistical software (Addinsoft, Paris, France).

#### Compound identification

The identity of compounds that were found to be significant in class separation found by LC-MS analyses were confirmed by LC-MS/MS performed with the QqQ mass analyser using the equipments described above. Experiments were repeated with chromatographic conditions identical to those described for the primary analysis. Ions were targeted for collision induced dissociation (CID) fragmentation in the QqQ based on the previously determined accurate mass and RT determined with the LC-MS apparatus. Comparison of the structure of the proposed compound with the fragments obtained confirmed the identity. Accurate mass data and isotopic distributions for the precursor and product ions were studied and compared to spectral data of reference compounds, if available, obtained under identical conditions for final confirmation (HMDB, METLIN).

#### Quantitative analyses of free fatty acids and cholesterol sulfate by LC-MS

To assess the major FFA and CHS quantitatively, 1:1 serial dilutions of a starting solution containing µmole/L of authentic FA C16:1, FA C18:1, FA C18:2, and FA C24:0, and 10 µmol/L of CHS were performed. The standard solutions were analysed with the LC-MS method described above. The limit of detection (LOD) and the limit of quantification (LOQ) were calculated based on the IUPAC guidelines^43^. In particular, LOD was determined as the lowest concentration that had a signal-to-noise ratio (S/N) 3 times higher than the blank sample. The LOQ was defined as the lowest concentration at which the linearity started. Linearity was defined on the basis of the correlation coefficient (R). Moreover, accuracy, intraday and interday reproducibility, and recovery were determined by injecting the solution 50 and 5 µmol/L of standard FFA and CHS, respectively.

### Data availability

The datasets generated during and/or analysed during the current study are available from the corresponding author on reasonable request.

## Results

A total of twenty-eight SC samples were collected from 10 healthy volunteers (for cohort characteristics, see Supplementary Table S1). In particular, twenty-four SC samples were collected from forearms (ARM), chests (CHEST), and foreheads (HEAD) of 8 females, whereas four SC samples were obtained from ARM and HEAD of 2 males. The sampled skin sites covered a wide range of SG density and abundance of sebum. To eliminate the interference due to the superficial sebum, its excess was removed by absorption with filter paper before the SC sampling. Then, amounts of SC sampled from each skin area were assessed gravimetrically. Weights of sampled SC are reported in the Supplementary Table S1A. On average, while the weight of the SC sampled at the CHEST was not significantly different from the one determined at the ARM site, the SC sampled at the HEAD site was significantly higher than both ARM and CHEST (p = 0.002, and p = 0.001, respectively). Moreover, the SC weight determined at the ARM was positively correlated with the ones measured at the CHEST and HEAD sites, respectively (Supplementary Table S1B). Likely due to the sample size significance of correlations was not reached (p > 0.05). Nevertheless, results suggested that the amount of SC that detached by tape-stripping was partly inherent to the donor. Due to the significant differences of the weights of samples from the different body areas, normalization by the weight was applied to the following investigations. Lipid profiling of SC samples obtained from the ARM, CHEST and HEAD body areas was analysed by LC-MS and detected in negative ion mode. Representative chromatogram of the SC is reported in Fig. S1. The negative ion mode is elective for the detection of epidermal lipids in the SC crude extracts with no sample pretreatment apart from evaporation and concentration^30^. Moreover, the analytical conditions used allowed to specifically address the ceramide families and CHS, which are classes specific to the permeability barrier in the SC. The FFA lipid family is populated by several members shared by sebum and SC. Nevertheless, the chemical features such as chain length, number of double bonds (DB), and branching vary considerably between the two skin lipid domains. Collectively, FFA, CHS, and ceramides show the propensity to form deprotonated ions detectable in negative ion mode. In contrast, negative ionization of interfering substances deriving from the patches and skin is less likely to occur. Two replicates of each SC extracts were subjected to the analysis. As shown in Fig. S1, long chain FA (LCFA, chain length of C12-20) and CHS were eluted in the first part of the chromatogram, followed by very long chain FA (VLCFA, C > 20) and ultra-long chain FA (ULCFA, C ≥ 26)^44^ and ceramides that were eluted later on between 14 and 22 minutes. It was noted that lipids eluted at late RT, including sphingolipids, tended to form three pseudomolecular ions. The first one was [M-H]^−^ due to the proton loss, whereas the following two ions were assigned as [M + HCOO]^−^ and [M- + C2O4H2Na]^−^ formed, respectively, upon interaction with formate and formate salts with alkali metals in the mobile phase. Evidence of the formation of the above pseudomolecular ions is provided with the supplemental material (see further). Moreover, neutral loss (NL) of water yielding the [M-H-H_2_O]^−^ ion was a frequent occurrence among tentatively assigned sphingolipids. The acquired data files were subjected to naïve search of features with the MFE algorithm in the Mass Hunter data analysis software. Extensive statistical analysis was performed with the MPP software in order to recognize patterns of lipid expression in body areas characterized by different density of the sebaceous gland.

### Significance testing and principal component analysis

Following alignment and normalization, features were filtered by choosing the entities that were present in 100% of samples from any experimental group, such as ARM, CHEST, and HEAD, respectively. More than a thousand (1098) entities were found in the totality (100%) of SC samples. Differences between SC for all three groups were evaluated for individual metabolites using ANOVA (with p ≤0.05), calculated using MPP. ANOVA performed on the filtered features returned six hundred (600) entities that were significantly different in ARM, CHEST, and HEAD lipid extracts, of which 414 features had a fold change higher that 2 (FC ≥2). Tukey’s Honest Significance Difference (HSD) post Hoc test was applied to identify which entities were responsible for significant differences in the three groups. Summary of ANOVA and HSD post Hoc test is reported in the Supplementary Table S2.

**Figure 1:**
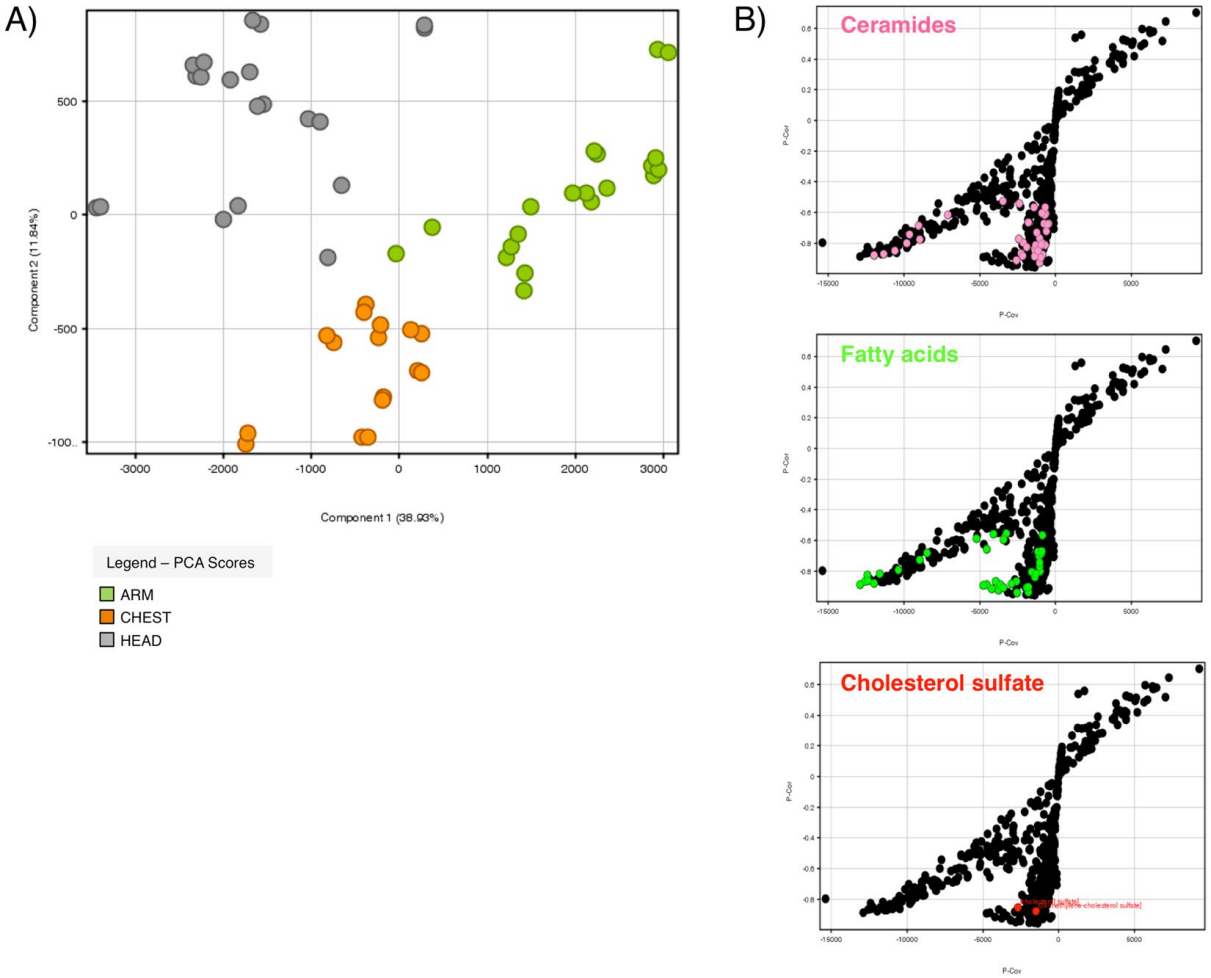
**(A)** Principal component analysis (PCA), and **(B)** C-C plots of the entities found in 100% samples of at least one condition among lipid extracts of ARM, CHEST, and HEAD stratum corneum (SC). Within the first two principal components (PC) 50.77% of the total variance was explained, with 38.93% in the first dimension and an additional 11.84% in the second dimension. The C-C plots show the P-Cor and P-Cov values of entities assigned as ceramides (pink dots), free fatty acids (green dots) and cholesterol sulfate related compounds (red dots) among the entities detected in 100% samples in at least one condition (black dots).

A graphical representation of the sample similarities and dissimilarities was obtained by PCA using the MPP software. Within the first two principal components (PCs), 50.77% of the total variance was explained, with 38.93% in the first dimension (PC 1) and an additional 11.84% in the second dimension (PC 2) (Fig. 1A). PCA analysis demonstrated to separate the three body sites making it possible to discriminate lipid extracts from SC sampled at different SG density. The loadings of the elements responsible for the separation of the different samples are shown in Fig. 1B. The distance of each element from the origin of the plot is an indication of the strength of the contribution of that element as a differentiator between body sites at different SG density. Accurate masses of features representing significant differences were searched against METLIN using the ID search tool. It emerged that several members of the ceramide family played a major role in the differentiation, together with FFA, CHS and a related compound, as reported in Fig. 1B. Positioning of the entities annotated as ceramides, FFA, and cholesterol sulfate related compounds in the C-C plots was visualized by highlighting dots with pink, green, and red colours, respectively.

### Fold changes and clustering

**Figure 2:**
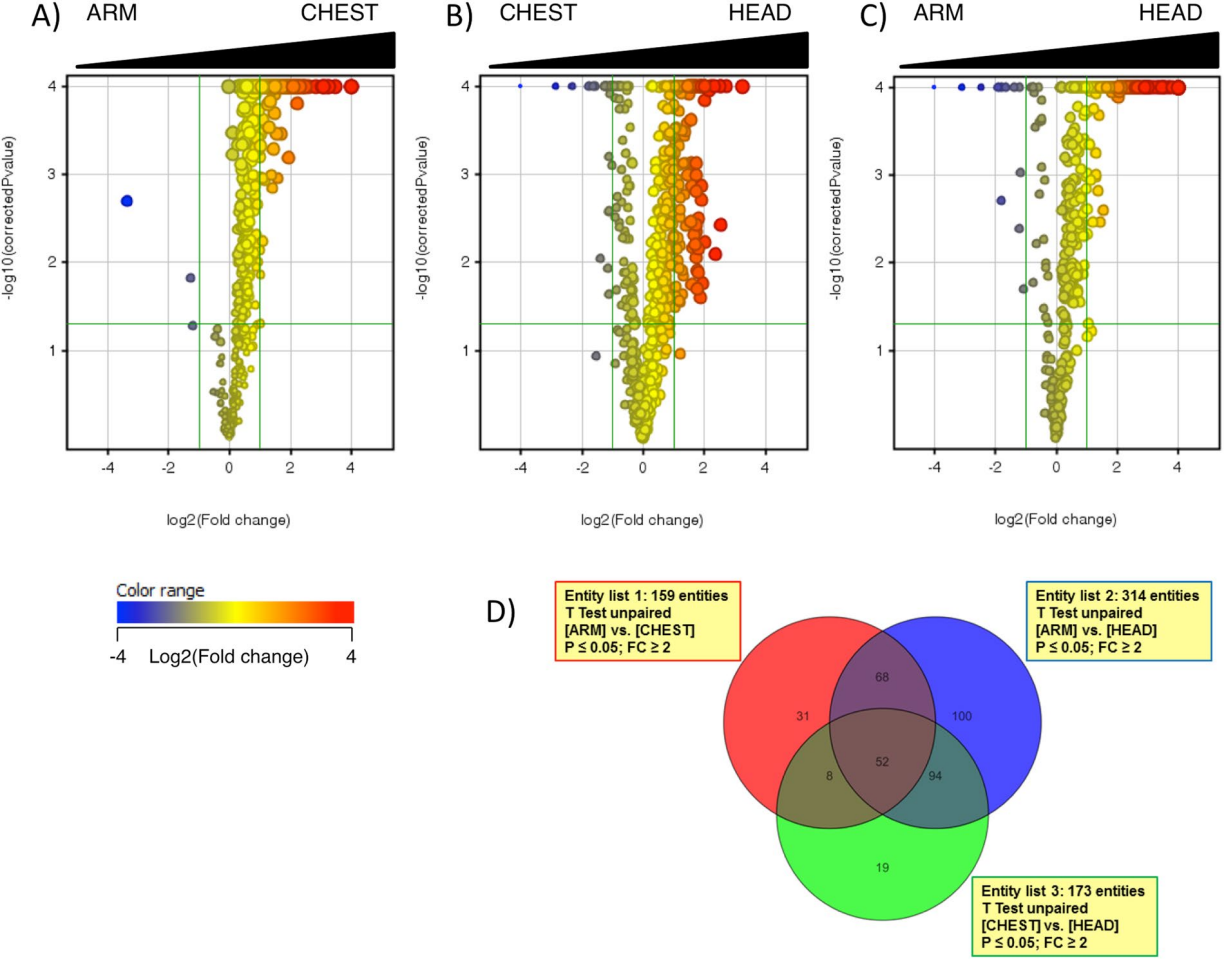
Volcano plots of entities found differently regulated in ARM, CHEST, and HEAD stratum corneum (SC). Comparison of relative abundance of entities between ARM and CHEST **(A)**, CHEST and HEAD **(B)**, ARM and HEAD **(C)** were performed by applying univariate significance test analysis to 600 entities found in the 100% of samples belonging to at least one condition. The direction from low-to-high SG density is depicted at the top of each volcano plot. The log2 of the fold-change (FC) values were plotted on the x-axis, whereas the −log10 of the t-test p-values were plotted on the y-axis. The vertical and the horizontal green lines marked the threshold of the FC and of the p-values with Bonferroni’s correction set at 2 and 0.05, respectively. The dots corresponding to each entity were coloured according to the FC, with red and blue colour indicating the highest and lowest FC values, respectively, for each pair-wise comparison, as depicted in the colour grade scale. Annotation of species found differently expressed is provided elsewhere in the results and following figures. Volcano plots returned 159, 173, and 314 entities that were expressed at a significantly different extent in the comparisons ARM vs. CHEST **(A)**, CHEST vs. HEAD **(B)**, ARM vs. HEAD **(C)**, respectively. **(D)** Eulero-Venn’s diagram depicting the number of entities that were commonly modified when comparing ARM to CHEST, CHEST to HEAD, and ARM to HEAD.

**Figure 3:**
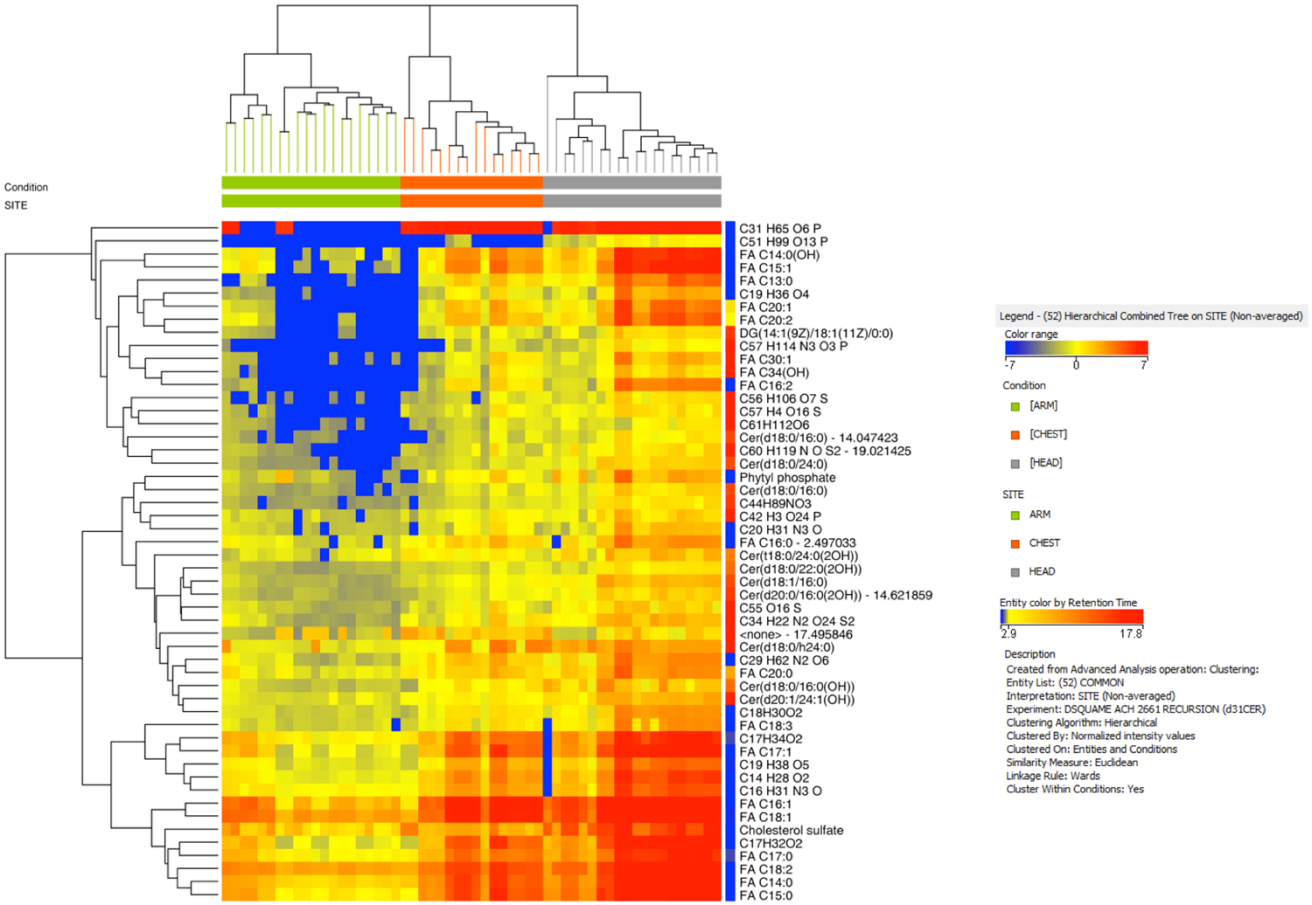
Hierarchical clustering of 52 entities that were commonly modified when comparing ARM to CHEST, CHEST to HEAD, and ARM to HEAD as reported in Fig. 2. The identity of annotated FFA and cholesterol sulfate was confirmed by comparison with RT and MS/MS spectrum of the respective authentic compounds. Ceramide annotations were confirmed upon generation of MS/MS spectra.

Differences between SC samples were evaluated by volcano plotting the 600 features for the following comparisons: ARM vs. CHEST; ARM vs. HEAD, and CHEST vs. HEAD. Comparisons were performed with t-test (with p < 0.05) with the Benjamini–Hochberg correction. As expected, volcano plots showed that the relative abundance of lipid metabolites was higher coincidentally with the higher SG density as shown in panels A, B, and C of Fig. 2 corresponding to the ARM vs. CHEST, ARM vs. HEAD, and CHEST vs. HEAD pair-wise comparisons, respectively. Venn’s diagram allowed for the selection of lipid metabolites that were significantly regulated due to the SG density. In particular, 52 species were commonly regulated when ARM lipidome was compared to CHEST and HEAD, and when CHEST lipidome was compared to HEAD (Fig. 2D). Cluster analysis was performed in order to organize either entities or groups of samples into clusters according to the site of sampling. Hierarchical clustering was performed by applying Pearson’s uncentered-absolute distance metric, complete linkage to the 52 lipid compounds common to the three groups of the Venn’s diagram. The heatmap in Fig. 3 illustrates the differences of expression of entities, including ceramides, monitored in the SC from different sites. The heatmap demonstrated clear clustering of lipids detected in ARM, CHEST and HEAD SC samples. The relative abundance of numerous FFA increased consistently with the increased density of the SG indicating that the sebum secretion absorbed by the SC greatly contributed to the abundance of FFA in the SC (see the paragraph below for details). Nevertheless, the major epidermal FFA, namely lignoceric and nervonic FA (FA C24:0 and FA C24:1n-9, respectively) were not included among the 52 entities commonly modulated in the pair-wise comparisons described above although FFA of epidermal origin were detectable under our experimental and analytical conditions, as detailed in the following paragraph. Consistent with the FFA abundance profile, we observed that the relative abundance of CHS and several members of the ceramide family was HEAD > CHEST > ARM. Altogether, these findings indicated an association between lipid markers of both sebaceous and epidermal lipids. The ceramide lipid class is large and complex in the SC. We attempted the identification of those ceramide members that were associated with changes in the lipid fingerprint of SC according to the SG density. In order to visualize the distribution of ceramides according to the SG density, a list of annotated ceramides was selected by the name within the filtering tool of the MPP software. The hierarchical clustering performed as described reported in Fig. S2 showed that the annotated ceramides distributed differently according to the SG density. Nevertheless, a general tendency to increase in their amount in the HEAD samples was observed. In order to confirm the identity of ceramides, MS/MS spectra were acquired. The excellent sensitivity of [M + H]^+^ ions in positive ion mode, together with the rich fragmentation spectra providing information of both the FA and the sphingoid base moieties in the ceramide molecule, makes it the preferred choice for identification purposes. Thus, MS/MS spectra were obtained by QqQ fragmentation in positive ion mode following elution in the same chromatographic conditions. MS/MS spectra and profiles of abundance of annotated ceramides that were affected differently in the three sites are reported in Supplementary Fig. S2. The ceramides presenting significantly different levels according to the SG distribution belonged to the main sphingolipid classes described in the human SC^6,18^. In particular, members of the Cer[NDS], Cer[ADS], Cer[NS], Cer[AS], Cer[NP], and Cer[AP] with a number of total carbon atoms ranging from 32 to 46 presented significantly higher levels in the SC sampled from sites at high SG density, regardless the size of both sphingoid base and N-acyl chain (Fig. S2). Cer[NS] and Cer[NDS] bearing FA with a chain length ranging between the 14 and 24 carbon atoms have been detected in both sebum and sweat in humans, with sebum presenting the highest levels of the two biofluids^45^. Conflicting results emerged between the latter study and a previous one^46^ suggesting that levels of ceramides can be regulated differently in sebum and SC. These findings pose the importance of assessing sebum excretion levels when sebum is the lipid matrix addressed in skin conditions associated with impaired SG activity^45,47^.

### Profiles of distribution of free fatty acids in the SC from forearms, chests, and foreheads

FFA are components of both sebum and SC. Nevertheless, the type and the distribution of specific FFA are characteristic of each lipid source. In particular, sebum is characterized by considerable amounts of FFA with chain length comprised between 14 and 18 carbon atoms, and prevalence of monounsaturated FA (MUFA) compared to other lipid sources. In contrast, epidermal FFA mainly consist of saturated carbon chains with a prevalent chain length varying between 24 and 26 carbon atoms^44,48–50^. Sapienic acid (FA C16:1n-10) is the isomer of FA C16:1 with a DB in delta 6 position that is peculiar to sebum. In contrast, epidermal lipids contain MUFA with DB typically at position delta 9. In addition, MUFA are less abundant than other epidermal FFA. Sebum presents also appreciable amounts of FFA with an odd number of C-atoms. In particular odd-numbered FFA can have straight or branched chain. However, branching can involve also even-numbered FFA, although at a lesser extent. Commonly, branches of sebaceous FFA have been described to have iso- or anteiso- isomerism. In agreement with the basic retention properties of FA methyl esters (FAME) in RP-HPLC, also RT of straight chain FFA increased with increasing carbon chain length. Unsaturated FFA eluted faster than the corresponding saturated FFA (SFA) with the same chain length the more DB they contained. A DB equalled with about two C-atoms in the RP-HPLC separation^51,52^. As shown in Fig. S3, FFA detected in the SC extracts covered the typical composition of both SC and sebum. Distribution of SFA with a number of C-atoms ranging from 12 and 26, and MUFA with a chain length ranging from 14 to 24 C-atoms, along with the FA 18:2 were assessed in the three body sites, namely ARM, CHEST, and HEAD. The profiled FFA were identified by means of authentic standards. Nevertheless, due to the poor separation of isomer FFA in the used LC-MS system, it is likely that co-elution of isobaric species occurred. Relative amounts of isobaric compounds were calculated regardless to their isomeric composition. Several FFA showed a significant difference in their abundance in the three sites. Regulation and statistical significance (ANOVA with Tukey’s HSD Post Hoc test analysis) of the differences between the categories with a confidence interval of 95%) are reported in the Supplementary Table S3.

### Quantitative analysis of principal free fatty acids in the SC and cholesterol sulfate by LC-MS

**Figure 4:**
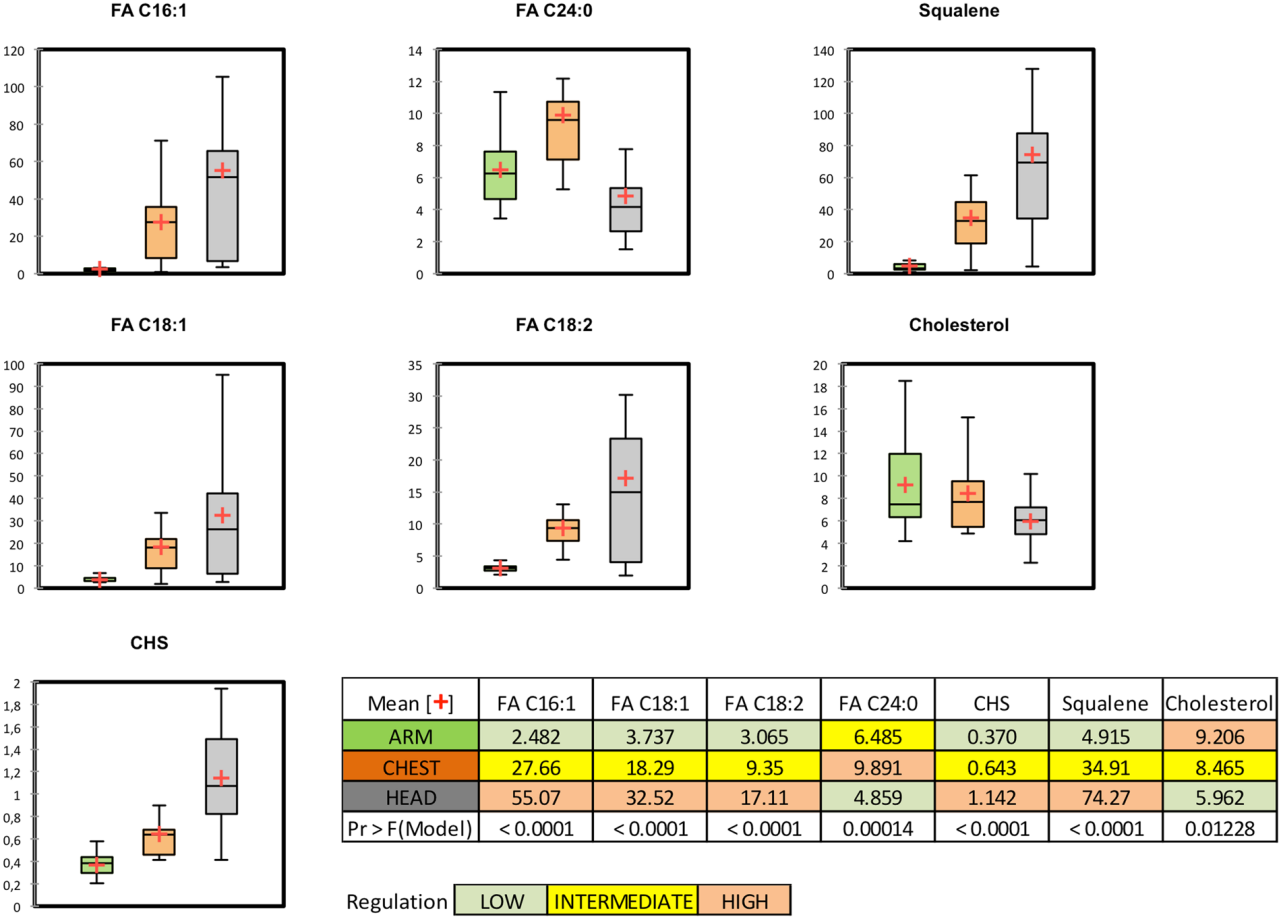
Results of the quantitative assessments of biomarkers of sebum secretion and lipids of the epidermal permeability barrier in ARM, CHEST, and HEAD SC lipid extracts. FFA and CHS were quantified by LC-MS, whereas amounts cholesterol and squalene were assessed by GC-MS. Box plots report concentration expressed as nmol/mg SC in ARM (green box), CHEST (orange box), and HEAD (grey box). The table summary included in the figure reports the mean concentration marked with a red cross in the respective box plot and the significance assessed with ANOVA.

To assess CHS levels and the amount of the major FFA characteristic of sebum and epidermal lipid barrier lipids, such as FA C16:1, FA C18:1, FA C18:2, and FA C24:0, a quantitative method based on the RP-HPLC separation and MS detection in negative ion mode was set-up. Linearity, LOD, LOQ, and recovery form the matrix were assessed for the authentic standards of the target analytes. The method was optimized to quantify FA C16:1, FA C18:1, FA C18:2, and FA C24:0 and CHS in the SC from ARM, CHEST, and HEAD. The FFA and CHS were quantified against the deuterated internal standards d14-PoA, and d7-CHS, respectively. The defined conditions demonstrated satisfactory linearity, reproducibility, recovery, sensitivity and LOQ as shown in Table S4. In order to normalize results to the weight of sampled SC, the quantitative amounts were divided by the sample mg. The concentrations of target FFA quantitated in the SC were consistent with the relative percentages observed for ARM, CHEST, and HEAD in the untargeted approach. As demonstrated by the quantitative results reported in Fig. 4 as nmol/mg SC, the sebum specific FFA, such as FA C16:1, increased in the order ARM < CHEST < HEAD. Similarly, FA C18:1, and FA C18:2 increased significantly in CHEST and HEAD, compared to ARM. In contrast the concentration of FA C24:0, a FFA peculiar of the epidermal lipid matrix, where it reaches higher amounts, demonstrated to be significantly reduced in HEAD SC compared to ARM SC. Interestingly, concentration of CHS, a derivative of cholesterol associated with epidermal lipid metabolism, increased in the SC in the order ARM < CHEST < HEAD, consistently with sebum lipid markers as indicated in the clustering analysis.

### Quantitative analysis of cholesterol and squalene in the SC by GC-MS

To corroborate the differences observed in the relative amounts of epidermal lipids and sebaceous lipids according to the SG distribution, respective biomarkers, namely cholesterol and squalene, were quantitatively determined by GC-MS on aliquots of the same SC extracts previously analysed by LC-MS. Quantitative results are reported as nmol of cholesterol and squalene for mg SC in Fig. 4 together with the LC-MS quantitative analyses of FFA and CHS. ANOVA demonstrated that concentrations of cholesterol decreased significantly moving from SG-poor toward SG-rich areas. In contrast, significant increase in the squalene concentration was observed when HEAD was compared to ARM and to CHEST sites.

## Discussion

The skin is a largely complex organ at both architectural and molecular level. Additionally, microbiome colonization and exposure to environmental factors further contribute to intricate the inherently complex lipid chemistry of skin^53^. Lipids are essential for several functions deployed by skin, being the outmost boundary with the external environment. Analysis of SSL from different regional areas can generate new hypotheses in the skin physiology and pathology. We have developed an analytical approach to investigate the skin lipid chemistry in body areas with different density of the SG with chemometric tools. Taking into account the two major sources of SSL, such as sebum and the SPB lipid matrix, we observed that the skin chemistry varied spatially along with the abundance of SG. Lipids specific to the epidermal compartment, including CHS, were significantly increased consistently with markers of the SG secretion, such as squalene and FA C16:1. In contrast, cholesterol and FA C24:0, which are found mainly in the lipid matrix of the SC present a profile of expression that is unrelated to the progressive increase of the SG density. CHS is generated from cholesterol by cholesterol sulfotransferase and desulfated back to cholesterol in the outer epidermis. The ‘cholesterol sulfate cycle’ active in the epidermis is crucial in the processes that regulate differentiation, SPB functions, and desquamation^54,55^. CHS is an amphipathic molecule expressed at relatively high concentration in the human epidermis, particularly in the granular layer. CHS functions as a regulatory molecule in the development of the epidermal barrier and in the differentiation of keratinocytes^56–58^. Amounts of ceramides, which are the major components of the SPB, proved to be influenced by the SG density. The presented data also suggest that enzymes involved with the CHS and ceramide synthesis^59^ might be sensitive to the amounts of sebum at the skin surface related to the density of the SG, in physiological conditions or to both density and activity of the SG in pathological skin conditions.

Large scale skin lipidomics has demonstrated the wide intraindividual variation of SSL according to the regional body area in humans^36^. Cheek, forehead, pectoral and shoulder regions presented a distinctive lipid profile compared to any other body site due to the sebaceous fingerprint mostly related to the higher levels of triglycerides and diglycerides^36^. Our analytical conditions were chosen in order to detect optimally the lipids of the epidermal barrier, such as ceramides and CHS, together with FFA. Abundance of specific members of the FFA family is a hallmark of the SPB and sebum lipid matrices. In our setting FFA accounted for the most distinctive signature of the sebaceous lipids. Our results were consistent with observations of the dramatically different structural organizations of lipid lamellae derived from corneocytes from the leg and the foreheads where the sebum content was detected at low (1 µg/cm^2^) and high (200 µg/cm^2^) level, respectively^60^. By means of Raman spectroscopy it has been shown that lipid content was increased in the dermis of SG-rich skin compared to SG-poor skin, demonstrating absorption of sebum components through epidermis and the underneath tissue^61^.

Although it was expected to find different chemical fingerprints due to the knowledge that skin features are not homogeneous across the body surface, this is the first evidence that amounts of the sebum lipids, due to higher density of the SG, impact the SC lipid metabolism and condition the expression of epidermal lipids. Changes in the sebum excretion rates have been associated with several skin disorders such as acne, seborrhoeic dermatitis, rosacea and atopic dermatitis^35,45,62,63^. Thus, perturbations of the SG activity occurring in pathological conditions superimpose to the density of the SG in determining the overall levels of sebum at skin surface. The role of the microbiota in the dialogue between sebum and skin permeability barrier has not been addressed in our design. Regions with a high density of the SG, such as the upper trunk and face, favour the growth of lipophilic microorganisms such as P. Acnes and M. Furfur^64^. The biochemical environment of the skin surface is essential for the microbial colonization, thus SSL can shape skin microbiome and immune surveillance and, in turn, be modelled by microbiome metabolism^65,66^. Further studies that elucidate the association between the site-specific lipidome with microbiome are needed to clarify their interplay. Revealing the impact of inherent SG secretion on the composition and organization of lipids in the SPB may be essential to understand the role of sebum in defining skin health. On one hand, sebum plays a crucial pathogenic role in skin dermatoses such as acne and seborrhoeic dermatitis, on the other hand it is little defined how sebum impacts the SC organization in these pathological conditions. Analogously, the knowledge on the role played by the SG density and/or activity in skin disorders that involve the SPB, such as psoriasis and atopic dermatitis, is rather limited. This study, together with the recent developments in the field of skin lipidomics, opens new opportunities to investigate simultaneously SG and SPB lipids in SC bearing together information arising from the two lipid domains.

## Electronic supplementary material

Supplementary information
