# Supplementary material for: Influence of the sebaceous gland density on the stratum corneum lipidome
Source: Sci Rep. 2018 Jul 31;8:11500. doi: 10.1038/s41598-018-29742-7 (PMC6068117; doi:10.1038/s41598-018-29742-7)
Supplement: Supplementary file 1 — Supplementary information [file 41598_2018_29742_MOESM1_ESM.docx]

**Supplementary information**

**INFLUENCE OF the sebaceous gland density on the STRATUM CORNEUM LIPIDOME**

^1#^Matteo Ludovici, ^1,2#^Nina Kozul, ^2^Stefano Materazzi, ^2^Roberta Risoluti, ^1^Mauro Picardo, ^1^*Emanuela Camera

^1^Laboratory of Cutaneous Physiopathology, San Gallicano Dermatological Institute IRCCS, Rome, Italy; ^2^Department of Chemistry, University of Rome “Sapienza”, Rome, Italy

^#^Contributed equally

*Corresponding Author

**Supplementary Figure S1**


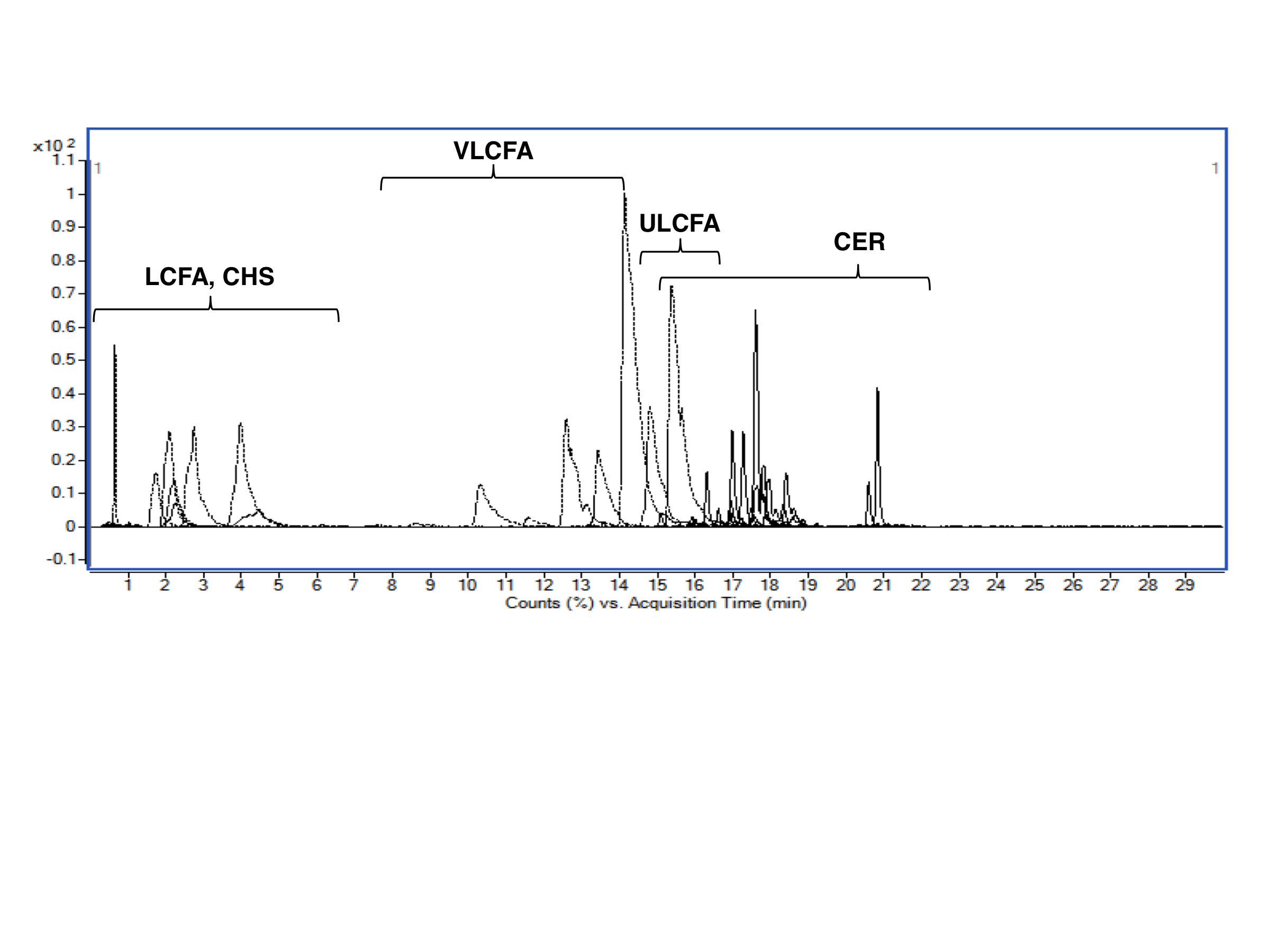


**Figure S1.** Merged extracted ion currents (EICs) of the [M-H]^-^ ions of free fatty acids (FFA) and cholesterol sulfate (CHS) (dashed line), and of the [M+HCOO]^-^ ions of selected ceramides (CER) (full line) of a forearm (ARM) SC crude lipid extract separated by RP-HPLC (Kinetex C8 column, Phenomenex) and detected by (-)ESI-TOF/MS (G6220A, Agilent Technologies). LCFA = long chain fatty acids (number of C-atoms 12-20), VLCFA very long chain fatty acids (number of C-atoms >20), ULCFA =ultra-long chain fatty acids (number of C-atoms ≥26).

**Supplementary Figure S2**


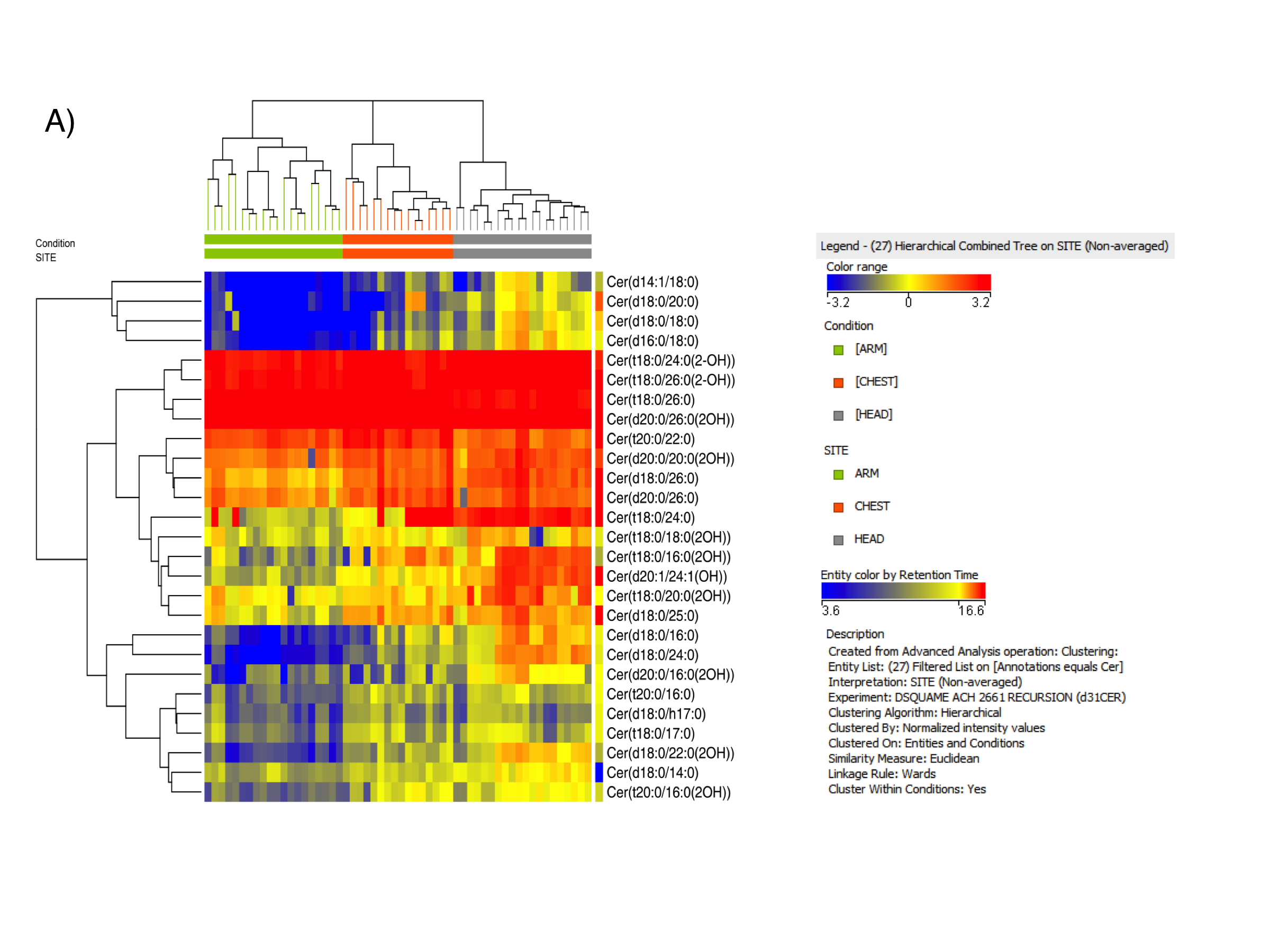


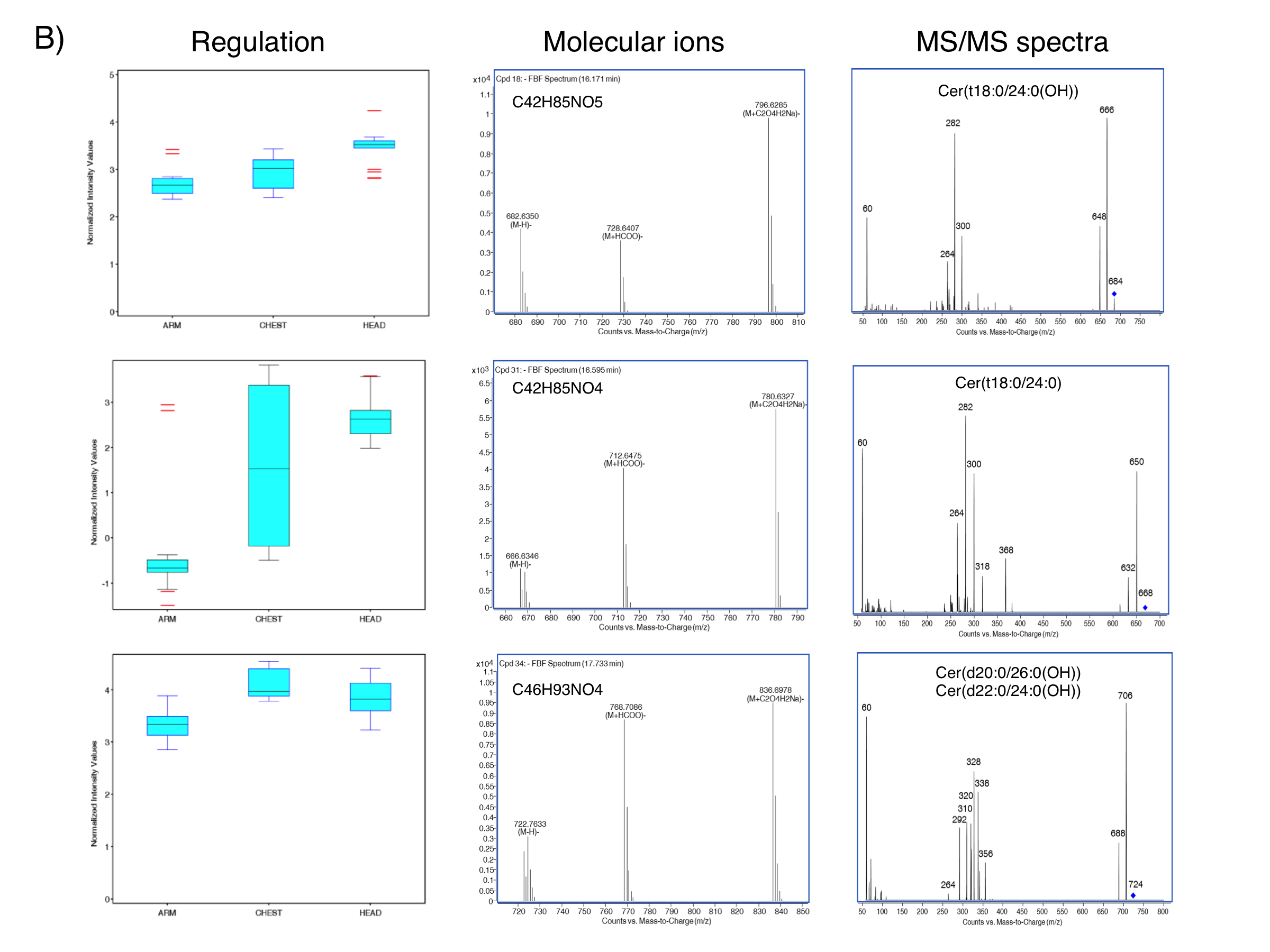


**Figure S2.** (A) Hierarchical clustering of annotated ceramides. (B) Regulation (box plots), molecular ions and generated elemental formula for the neutral compound (accurate mass as detected by TOF-MS), and MS/MS spectra (obtained upon product ion scanning with the QqQ-MS) of representative ceramide species in the hierarchical clustering.

**Supplementary Figure S3**


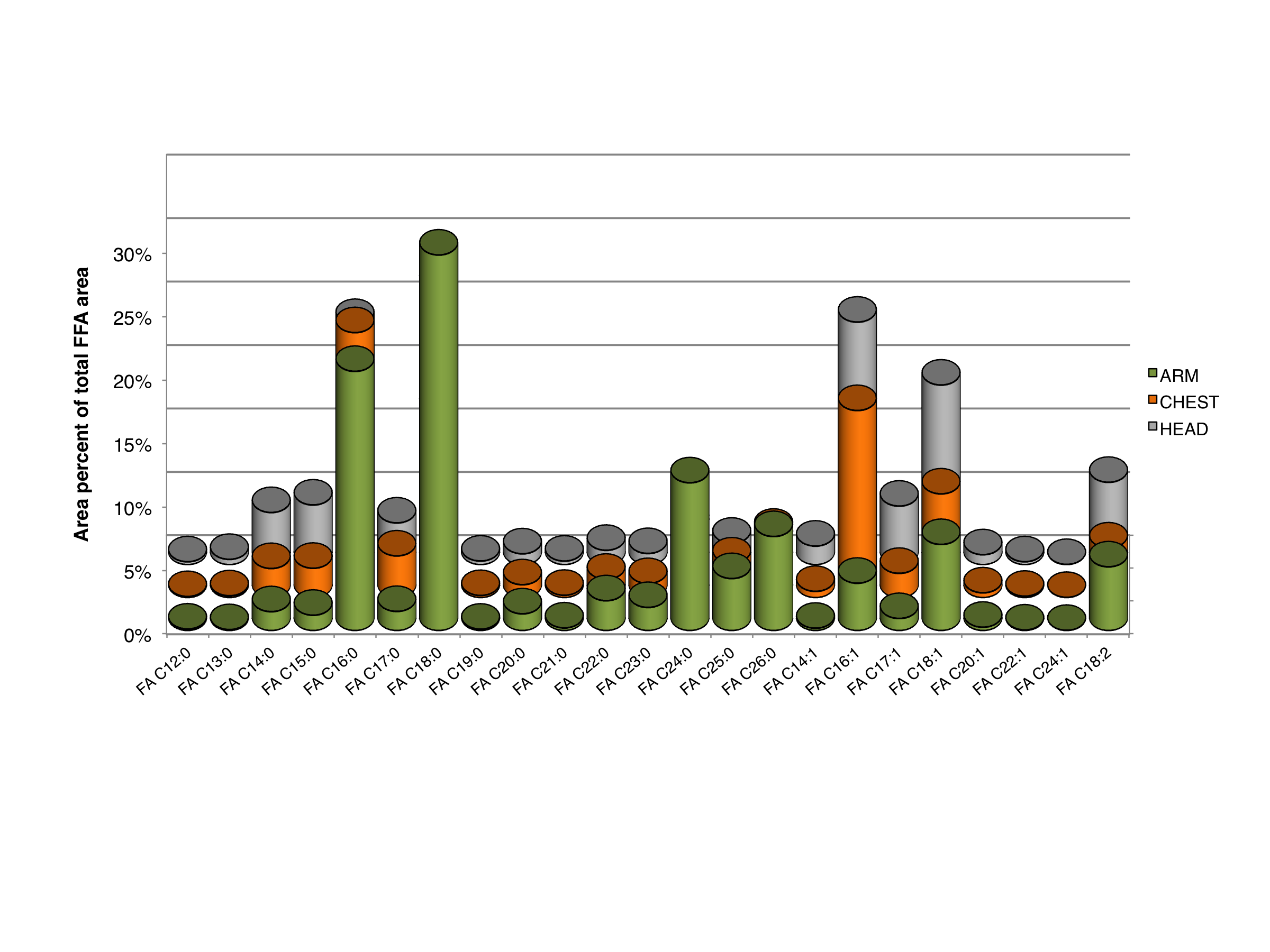


**Figure S3.** A) Profiles of distribution of free fatty acids (FFA) in the stratum corneum (SC) sampled from ARM, CHEST, and HEAD. Relative abundance of FFA was reported as percentage of peak area of the individual FFA on the total FFA area.

Supplementary tables

**Supplementary Table S1.** Demographics and sample description.

1. Demography of the stratum corneum (SC) donors

| ID | Gender | *Age | **ARM | **CHEST | **HEAD |
| --- | --- | --- | --- | --- | --- |
| SC01 | F | > 40 | 0.73 | 0.57 | 1.52 |
| SC02 | F | > 40 | 0.94 | 1.13 | 1.58 |
| SC03 | F | > 40 | 0.52 | 0.37 | 0.62 |
| SC04 | F | < 40 | 0.82 | 1.07 | 1.43 |
| SC05 | F | < 40 | 0.49 | 0.80 | 1.17 |
| SC06 | F | < 40 | 0.54 | 0.71 | 0.86 |
| SC07 | F | < 40 | 0.82 | 0.61 | 1.10 |
| SC08 | F | < 40 | 0.77 | 1.02 | 2.56 |
| SC09 | M | > 40 | 1.40 | - | 2.01 |
| SC10 | M | < 40 | 0.84 | - | 1.71 |
|  |  | AVERAGE | 0.787 | 0.785 | 1.456 |
|  |  | SD | 0.264 | 0.270 | 0.565 |
|  |  | RSD % | 33.6 | 34.4 | 38.8 |
|  |  | *Age in years; **SC weight in mg. | | | |

1. Matrix of Pearson’s correlations and p values for comparisons among SC weights in ARM, CHEST, and HEAD

| Correlation matrix (Pearson): | | |  |  |  |  |
| --- | --- | --- | --- | --- | --- | --- |
| Variables | ARM | CHEST | HEAD |  |  |  |
| ARM | **1** | 0.602 | 0.590 |  |  |  |
| CHEST | 0.602 | **1** | 0.657 |  |  |  |
| HEAD | 0.590 | 0.657 | **1** |  |  |  |
| *Values in bold are different from 0 with a significance level alpha=0.05*   \| p-values: \|  \|  \|  \|  \|  \|  \| \| --- \| --- \| --- \| --- \| --- \| --- \| --- \| \| Variables \| ARM \| CHEST \| HEAD \|  \|  \|  \| \| ARM \| **0** \| 0.114 \| 0.073 \|  \|  \|  \| \| CHEST \| 0.114 \| **0** \| 0.076 \|  \|  \|  \| \| HEAD \| 0.073 \| 0.076 \| **0** \|  \|  \|  \| \| *Values in bold are different from 0 with a significance level alpha=0.05* \| \| \| \| \| \| \| | | | | | | |

**Supplementary Table S2.** ANOVA of the 600 entities resulted significantly different among the 1080 filtered in the data sets from the ARM, CHEST, and HEAD lipid extract analyses. Of the 600 entities, 414 features had a fold change higher that 2 (FC ≥2). Tukey’s Honest Significance Difference (HSD) post Hoc test was applied to identify the number of entities responsible for significant differences in the three groups.


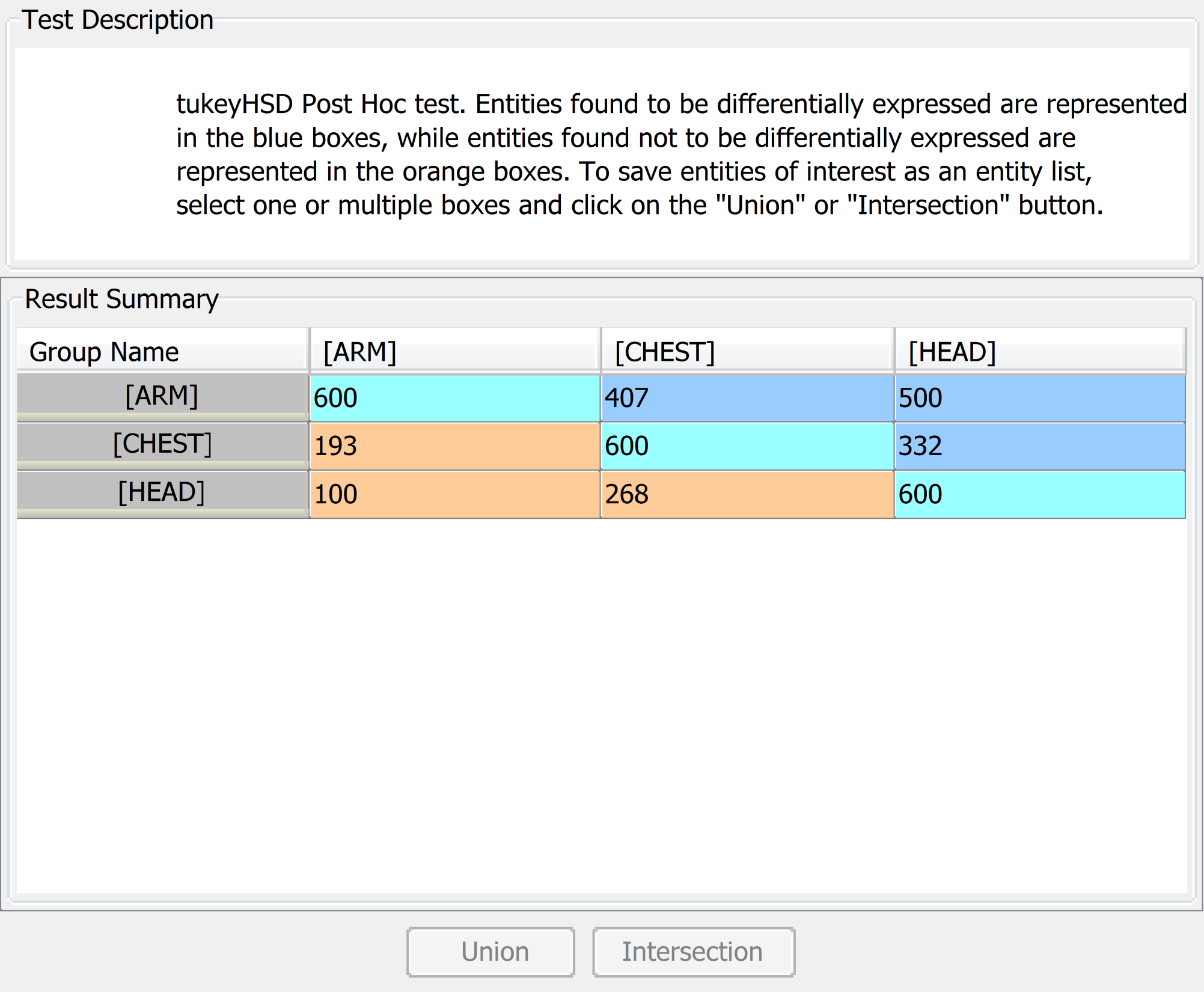


Output of the ANOVA performed on 600 entities with statistically different expression among the 1080 entities. Entities found to be differentially expressed between groups by Tukey’s HSD Post Hoc test are represented in blue boxes, while entities found not to be differentially expressed are represented in orange boxes.

**Supplementary Table S3.** Comparisons of relative abundance of free fatty acids (FFA) in ARM, CHEST, and HEAD and significance of differences following ANOVA and Tukey’s post-hoc HSD.

Relative abundance of FFA in SC reported as ($FFA peak area)/(total FFA peak area)$

|  |  | ARM | CHEST | HEAD | Pr > F | Significant |
| --- | --- | --- | --- | --- | --- | --- |
| SFA | FA C12:0 | 1.05E-03 | 6.96E-04 | 2.11E-03 | **0.046** | Yes |
|  | FA C13:0 | 5.74E-04 | 1.26E-03 | 4.01E-03 | **0.000** | Yes |
|  | FA C14:0 | 1.47E-02 | 2.28E-02 | 4.09E-02 | **0.000** | Yes |
|  | FA C15:0 | 1.19E-02 | 2.30E-02 | 4.68E-02 | **0.000** | Yes |
|  | FA C16:0 | 2.04E-01 | 2.09E-01 | 1.89E-01 | 0.576 | No |
|  | FA C17:0 | 1.49E-02 | 3.27E-02 | 3.27E-02 | 0.298 | No |
|  | FA C18:0 | 2.96E-01 | 2.44E-01 | 1.21E-01 | **0.010** | Yes |
|  | FA C19:0 | 9.73E-04 | 1.50E-03 | 2.66E-03 | **0.000** | Yes |
|  | FA C20:0 | 1.30E-02 | 9.97E-03 | 8.37E-03 | **0.020** | Yes |
|  | FA C21:0 | 1.92E-03 | 1.66E-03 | 2.62E-03 | **0.015** | Yes |
|  | FA C22:0 | 2.33E-02 | 1.43E-02 | 1.11E-02 | **0.001** | Yes |
|  | FA C23:0 | 1.80E-02 | 1.09E-02 | 8.61E-03 | **0.000** | Yes |
|  | FA C24:0 | 1.16E-01 | 5.22E-02 | 2.92E-02 | **0.000** | Yes |
|  | FA C25:0 | 4.08E-02 | 2.71E-02 | 1.68E-02 | **0.000** | Yes |
|  | FA C26:0 | 7.39E-02 | 4.98E-02 | 1.67E-02 | **0.000** | Yes |
| MUFA | FA C14:1 | 1.68E-03 | 4.75E-03 | 1.45E-02 | **0.000** | Yes |
|  | FA C16:1 | 3.71E-02 | 1.47E-01 | 1.91E-01 | **0.011** | Yes |
|  | FA C17:1 | 9.34E-03 | 1.89E-02 | 4.59E-02 | **0.000** | Yes |
|  | FA C18:1 | 6.77E-02 | 8.16E-02 | 1.41E-01 | **0.002** | Yes |
|  | FA C20:1 | 2.46E-03 | 3.57E-03 | 7.65E-03 | **0.000** | Yes |
|  | FA C22:1 | 2.91E-04 | 9.17E-04 | 2.12E-03 | **0.000** | Yes |
|  | FA C24:1 | 7.39E-06 | 2.05E-06 | 2.54E-06 | 0.086 | No |
| PUFA | FA C18:2 | 5.00E-02 | 3.90E-02 | 6.47E-02 | **0.001** | Yes |

SFA= Saturated FA; MUFA= Monounsaturated FA; PUFA= Polyunsaturated FA

| LOW | INTERMEDIATE | HIGH |
| --- | --- | --- |

**Supplementary Table S4.** Performance of the quantitative method optimized to determine selected FFA and CHS.

|  | ^#^a | ^#^b | R | *Accuracy | *RSD % intraday | *RSD % interday | **LOD | **LOQ | Recovery  % |
| --- | --- | --- | --- | --- | --- | --- | --- | --- | --- |
| CHS | 1.1 | 0.048 | >0.999 | 97.85 | 11.4 | 11.32 | 0.02 | 0.045 | 92 |
| FA C16:1 | 8.9 | -0.008 | >0.999 | 98.6 | 4.38 | 5.71 | 3.46 | 4.02 | 99 |
| FA C18:1 | 13.1 | 0.129 | >0.999 | 97.8 | 4.56 | 6.91 | 4.84 | 5.79 | 99 |
| FA C18:2 | 12.5 | 0.029 | >0.999 | 99.6 | 2.68 | 5.18 | 3.12 | 4.15 | 106 |
| FA C24:0 | 92.6 | 0.499 | >0.999 | 97.3 | 4.4 | 5.85 | 0.36 | 0.87 | 98 |

^#^Coefficients of the equation $y=ax+b$, where x is the concentration in µmol/L, and y is the $\frac{analyte area}{internal standard area}$ ratio.

R=Correlation coefficient

*Determined on the 50 µmol/L and 5 µmol/L concentrations of FFA and CHS solutions, respectively

**expressed as µmol/L
